# Recognition of ZnT8, Proinsulin, and Homologous MAP Peptides in Sardinian Children at Risk of T1D Precedes Detection of Classical Islet Antibodies

**DOI:** 10.1155/2016/5842701

**Published:** 2015-12-28

**Authors:** Magdalena Niegowska, Daniela Paccagnini, Carla Mannu, Clara Targhetta, Marco Songini, Leonardo A. Sechi

**Affiliations:** ^1^Department of Biomedical Sciences, University of Sassari, 07100 Sassari, Italy; ^2^Centre for the Treatment of Complications of Diabetes, Hospital “G. Brotzu”, 09134 Cagliari, Italy

## Abstract

As numerous studies put in evidence the increasing incidence of type 1 diabetes (T1D) in children, an early diagnosis is of great importance to define correct treatment and diet. Currently, the identification of classical islet autoantibodies is the primary biomarker for diagnosis in subjects at risk, especially in pediatric patients. Recent studies suggest that detection of antibodies against ZnT8 protein in preclinical phase can predict the development of T1D. We previously demonstrated a significant association of* Mycobacterium avium* subspecies* paratuberculosis* (MAP) with T1D in adult Sardinian patients. To enforce this finding, we investigated the presence of antibodies against ZnT8 and proinsulin (PI) with respective homologous epitopes: MAP3865c_133–141_/ZnT8_186–194_, MAP3865c_125–133_/ZnT8_178–186_, MAP2404c_70–85_/PI_46–61_, and MAP1,4*α*gbp_157–173_/PI_64–80_, in 23 children at risk for T1D, formerly involved in the TRIGR study, and 22 healthy controls (HCs). Positivity to anti-MAP and homologous human peptides was detected in 48% of at-risk subjects compared to 5,85% HCs, preceding appearance of islet autoantibodies. Being MAP easily transmitted to humans with infected cow's milk and detected in retail infant formulas, MAP epitopes could be present in extensively hydrolyzed formula and act as antigens stimulating *β*-cell autoimmunity.

## 1. Introduction

Type 1 diabetes (T1D) is an autoimmune disease with increasing incidence in youth worldwide [1], characterized by impaired glucose tolerance progressing to hyperglycemia, which results from T-cell mediated pancreatic *β*-cell destruction and from a simultaneous production of antibodies (Abs) against islet cells. This process starts in early infancy and can occur over many years during childhood without developing clinical symptoms. The primary biomarkers for diagnosis in subjects at risk involve identification of Abs to islet cell antigens (ICA), glutamic acid decarboxylase 65 (GADA), tyrosine phosphatase-related insulinoma-associated 2 molecule (IA-2), and insulin (IAA), among which anti-(pro)insulin Abs appear at a younger mean age [2, 3] and are common in children progressing rapidly to overt diabetes [4]. Recently, zinc transporter 8 (ZnT8) protein has been described as a novel T1D autoimmunity target preceding clinical symptoms of the disease and increasing the sensitivity of detection when analyzed along with other T1D Abs [5]. Both proinsulin (PI) and ZnT8 contain amino acid sequences homologous to proteins of* Mycobacterium avium* subspecies* paratuberculosis* (MAP), a bacterium putatively associated with T1D [6–10].

MAP is the causative agent of Johne's disease in livestock ruminant species and has been associated with Crohn's disease in humans [11, 12]. As infected animals shed MAP in feces and at low concentrations in milk, the bacterium, able to survive pasteurisation process [13–16], can be transmitted with contaminated dairy products. According to numerous reports, MAP was detected in up to 3,6% of retail milk and cheese [17–19] and in 35% of infant powdered milk samples from seven European countries [20]. In light of suggestions that exposure to complex foreign proteins and bovine insulin early in life may increase the risk of *β*-cell immunity [21–26], the TRIGR study evaluated whether weaning to an extensively hydrolyzed formula reduces the risk of T1D in infants with HLA-conferred susceptibility and a first-degree relative affected by autoimmune diabetes [27]. The trial involved 2159 subjects recruited in 15 developed countries and randomly grouped to be weaned to a conventional cows' milk-based formula or a casein hydrolysate. Despite detection of two or more islet autoantibodies in 13,4% of children in the hydrolyzed formula group, a similar result was obtained for infants weaned to the conventional formula (11,4%), confirming that the formula type has no role in preventing or delaying the onset of autoimmune diabetes.

Following our previous outcomes hypothesizing a possible role of MAP as an environmental agent in T1D onset [7, 10], in the present study, we analyzed plasma samples of Sardinian children enrolled in the TRIGR study for the presence of Abs against selected MAP peptides and homologous ZnT8 and PI fragments to identify possible biomarkers for early prediction of T1D development towards overt clinical disease. Moreover, peptides homologous to the human ZnT8 protein derived from* Helicobacter pylori* (HP) were assessed in order to achieve a picture of seroreactivity to a pathogen not associated with T1D and a possible cross-reaction with anti-MAP Abs.

## 2. Materials and Methods

### 2.1. Subjects

In the framework of TRIGR study, 23 Sardinian children at risk of T1D (i.e., with a first-degree relative affected by T1D; mean age 2,8 ± 2,7 years) attending the Department of Diabetes, St. Michele Hospital of Cagliari, Italy, were enrolled in the study and followed in time in order to identify a correlation between the presence of Abs and the onset of diabetes. The first blood samples were collected at birth with further annual collections up to 10 years, giving in total 139 samples over the period of 2002–2012 with the mean age 6,65 ± 2,74 years at the end of follow-up. In addition, all at-risk subjects (ARS) were analyzed for the presence of ICA, GADA, IA-2, and IAA autoantibodies. We analyzed relative plasma samples for Abs against ZnT8, PI, and homologous MAP peptides. Reference control samples of age-matched healthy volunteers (HCs; *n* = 22, mean age 4,3 ± 2,1 years) were provided by the Tor Vergata University of Rome based on a single collection scheme. Blood samples were collected after obtaining written informed consent for all study participants from a parent or caretaker and plasma was separated by sedimentation. The study protocols were approved by the Ethical Committees of the University of Sassari, the St. Michele Hospital of Cagliari, and the Tor Vergata University of Rome, Italy, in accordance with the Declaration of Helsinki.

### 2.2. HLA Genotyping and Diabetes-Associated Autoantibody Assays

HLA genotype and levels of islet autoantibodies (ICA, IAA, GADA, and IA2A) were determined for all ARS as described elsewhere [27].

### 2.3. Peptides

Formerly identified immunodominant MAP-derived peptides of a cation transporter MAP3865c_133–141_ (LAANFVVAL) and MAP3865c_125–133_ (MIAVALAGL), a hypothetical protein MAP2404c_70–85_ (RGFVVLPVTRRDVTDV), and a glucan branching protein MAP1,4*α*gbp_157–173_ (GTVELLGGPLAHPFQPL) along with their respective homologs ZnT8_186–194_ (VAANIVLTV), ZnT8_178–186_ (MIIVSSCAV), PI_46–61_ (RGFFYTPKTRREAEDL), and PI_64–80_ (GQVELGGGPGAGSLQPL) [8, 28] were synthesized at >85% purity (LifeTein, South Plainfield, NJ 07080, USA) assessed by HPLC. Specificity of the peptides has been tested previously through competitive inhibition assay [8].

In addition, peptides J0I929_HELPX_1–11_ (MIIGGGVSGCA) derived from HP quinone oxidoreductase J0I929_HELPX protein (UniProtKB accession number J0I929) and T2T4W3_HELPX_99–105_ (AGIVLTV) derived from HP preprotein translocase subunit T2T4W3_HELPX (UniProtKB accession number T2T4W3), respective homologs of ZnT8_178–186_ and ZnT8_186–194_, were synthesized by the same manufacturer at >90% purity. All purified peptides were stored at −80°C in single-use aliquots as 10 mM solutions.

### 2.4. ELISA

Indirect ELISAs to detect Abs specific for MAP3865c/ZnT8, MAP1,4*α*gbp/PI, MAP2404c/PI, and HELPX/ZnT8 peptides were performed as described previously [28]. Data was normalized to a strongly positive control serum included in all assays with Abs reactivity set at 1.0 arbitrary units U/mL. Optimal cut-off points of 0,66 U/mL for MAP/ZnT8 and 0,76 U/mL for MAP/PI peptide pairs were identified based on the receiver operating characteristic (ROC) curves, setting specificity at 95%. Similarly, positivity cut-off points were determined for J0I929_HELPX_1–11_ (0,73 U/mL) and T2T4W3_HELPX_99–105_ (0,83 U/mL). Interassay coefficient of variation (CV) for the 8 peptide-based ELISAs ranged from 6,7% to 8,4%, whereas the intra-assay CV accounted for 2,8% upon 20 Abs reactivity tests. GraphPad Prism ver. 6.0 software was used for statistical analyses of the assay with levels of significance determined through Student's *t*-test (95% CI) or the Mann-Whitney *U* test for not normally distributed data.

## 3. Results

In the present study population, 10 out of 23 ARS (43,48%) were positive for anti-MAP1,4*α*gbp_157–173_ (M1) Abs, compared to 9,09% of HCs. The same prevalence among ARS was detected for the homologous PI_64–80_ (P1) peptide, but only in 13,64% of HCs.

**Figure 1:**
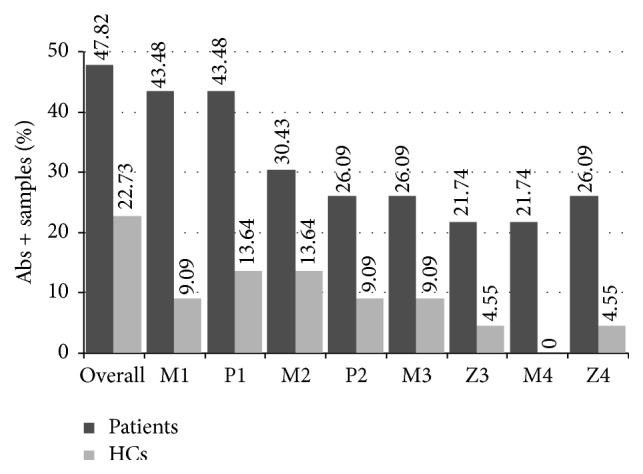
Prevalence of Abs against MAP, proinsulin, and ZnT8 homologous peptides in children at risk of T1D and healthy controls. Sera were tested in duplicate for their reactivity against plate-coated peptides: MAP1,4*α*gbp_157–173_ (M1); PI_64–80_ (P1); MAP2404c_70–85_ (M2); PI_46–61_ (P2); MAP3865c_133–141_ (M3); ZnT8_186–194_ (Z3); MAP3865c_125–133_ (M4); ZnT8_178–186_ (Z4). Percentage of children with Abs positivity to any of the analyzed peptides is indicated by the first column pair. Dark bars represent children at risk of T1D; light grey bars correspond to HCs.

Abs against MAP2404c_70–85_ (M2) were recognized by 7 ARS (30,43%) and 13,64% HCs, while 6 ARS (26,09%) and 9,09% of HCs were positive for its homolog PI_46–61_ (P2) (Figure 1).

Reactivity to MAP3865c_133–141_ (M3) was registered for 6 ARS (26,09%) and 9,09% of HCs, whereas 5 ARS (21,74%) were positive for the homologous ZnT8_186–194_ (Z3) peptide, comparing to 4,55% among HCs.

Anti-MAP3865c_125–133_ (M4) Abs were found in 5 ARS (21,74%) and in none of the HCs. Recognition of its homolog ZnT8_178–186_ (Z4) among the analyzed subjects accounted for 6 ARS (26,09%) and 4,55% of positivity for HCs. The frequencies of Abs here presented are noncumulative values based on evaluation whether positivity was detected or not during the follow-up.

**Figure 2:**
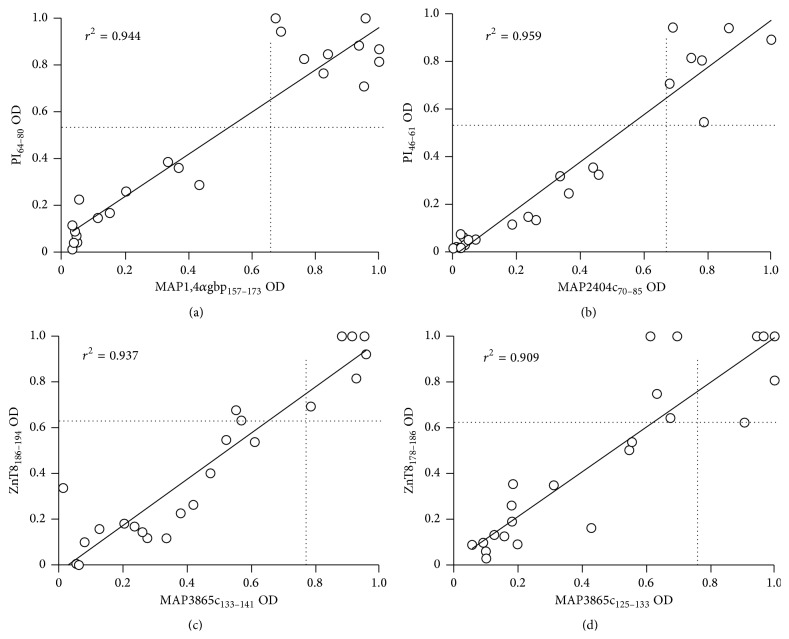
Correlation between Abs recognizing MAP and its homologous human epitopes in Sardinian children at risk for T1D. Correlations are shown between Abs against MAP1,4*α*gbp_157–173_/PI_64–80_ (a), MAP2404c_70–85_/PI_46–61_ (b), MAP3865c_133–141_/ZnT8_186–194_ (c), and MAP3865c_125–133_/ZnT8_178–186_ (d) in 23 at-risk children. Each circle represents Abs of one of ARS. The dotted lines indicate the cut-off for positivity used in each assay, as calculated by ROC analysis.

Upon screening for anti-islet autoantibodies, 6 out of 23 ARS were positive for ICA (26%), 3 for IAA and/or IA2 (13%), and 2 for GAD antibodies (8,69%) (Table 1). Overall, 47,82% of children (*n* = 11) reacted to any of the analyzed peptides compared to 22,73% of age-matched HCs. A high degree of correlation (*r* ^2^ > 0,9) was found between anti-MAP and the respective anti-PI/ZnT8 Abs titers (Figure 2).

**Figure 3:**
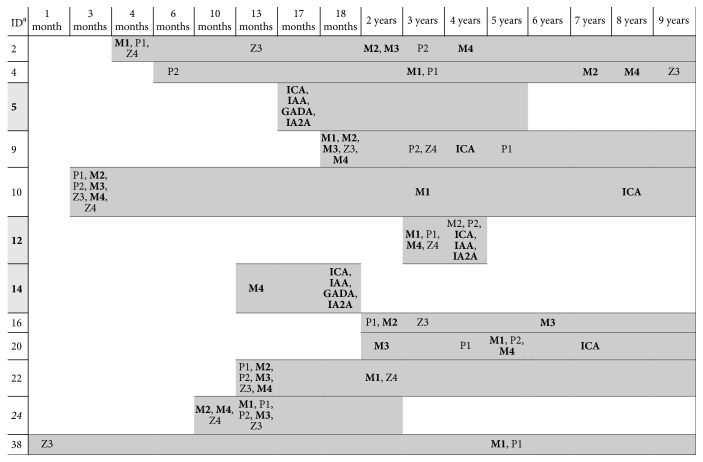
Age at detection of islet autoantibodies and immune reactivity to MAP, ZnT8, and PI homologous peptides in children at risk for T1D. Grey bars correspond to the time-point samples available for each child. ^a^: identity of children positive for the studied peptides or/and islet autoantibodies (*n* = 11); highlighted patients progressed to T1D at 5, 4, and 1,5 years of age, respectively; patient indicated in italics was lost during the follow-up.

Interestingly, of the 23 ARS followed in time, 5 were positive for all the peptides MAP3865c_133–141_, MAP3865c_125–133_, MAP2404c_70–85_, and MAP1,4*α*gbp_157–173_, ZnT8_186–194_, ZnT8_178–186_, PI_46–61_, and PI_64–80_ (Figure 3); in other 5 ARS, response to at least three peptides was registered. The highest reactivity among subjects positive for the analyzed epitopes was observed for MAP1,4*α*gbp_157–173_/PI_64–80_ peptide pair, registered for 10 at-risk children (91%); two of them additionally recognized Abs against MAP2404c_70–85_/PI_46–61_ homologs and anti-MAP3865c_125–133_/ZnT8_178–186_ Abs were found in one child. Four children presented Abs positivity against nonhomologous peptides MAP3865c_133–141_, MAP3865c_125–133_, MAP2404c_70–85_, ZnT8_186–194_, and PI_46–61_. The overlap in reactivity against homologous peptides among positive HCs (*n* = 5) accounted for 40% and regarded only two subjects with Abs directed against at least 4 epitopes: one HC recognized MAP1,4*α*gbp_157–173_/PI_64–80_ and MAP2404c_70–85_/PI_46–61_ peptide pair while the other one showed positivity to MAP3865c_133–141_/ZnT8_186–194_ and MAP2404c_70–85_/PI_46–61_ homologs. Nonhomologous reactivity was detected for MAP1,4*α*gbp_157–173_, PI_64–80_, MAP3865c_133–141_, ZnT8_178–186_, and MAP2404c_70–85_.

Five of the positive children presented autoimmunity against both the studied peptides and classical islet antibodies. Furthermore, positivity to our peptides not only appeared before detection of classical autoantibodies within 6 months of age (Figure 3), but was also maintained in time. Eventually, three ARS developed diabetes few years later demonstrating the importance of the studied peptides as potential preclinical biomarkers for diagnosis of T1D in subjects at risk. However, another child developed T1D even though antibody values were not significantly high, emphasizing the fact that T1D is determined by multiple factors contributing to disease manifestation.

**Figure 4:**
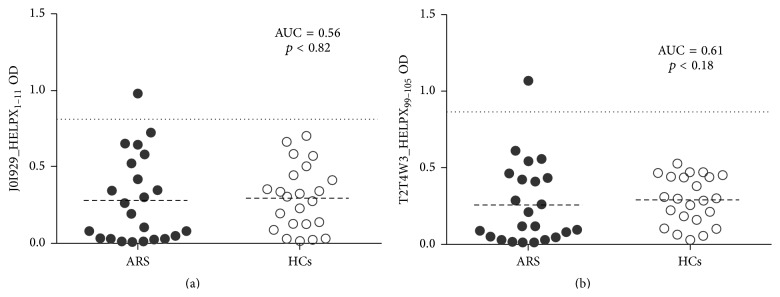
Distribution of anti-HP Abs levels measured by ELISA in T1D at-risk subjects and healthy controls. Sera of 23 ARS and 22 HCs were screened for Abs against J0I929_HELPX_1–11_ (a) and T2T4W3_HELPX_99–105_ (b). The dotted lines represent cut-off values calculated by ROC analysis and used to discriminate between positive and negative samples. Dashed lines indicate the respective mean OD values. Area under the curve (AUC) and *p* values are reported in the top-right corners.

Anti-HP Abs, either directed against J0I929_HELPX_1–11_ or T2T4W3_HELPX_99–105_, were detected in one of ARS (4,35%) and the results were not statistically significant (*p* < 0,82 and 0,18, resp.). No positive cases were observed among controls (Figure 4) and no correlation with progression to T1D or response to the analyzed peptides was found.

## 4. Discussion

As numerous studies put in evidence the increasing incidence of diabetes in children, an early diagnosis is of great importance to define a correct method of restraining disease development and to establish ulterior treatment. Although genetic susceptibility has been considered a key factor for T1D onset, less newly registered cases present genotypes of high or moderate risk [29–35]. Environmental factors seem to be critical in the pathogenesis of diabetes and include diet, infectious agents, and perinatal and psychosocial conditions that vary among countries [36]. Several intracellular pathogens have been studied as possible T1D triggers, but none of them was proved to cause the disease. Even though most researches focus on human enterovirus, our recent studies showed a strong association between MAP and type 1 diabetes [7–10].

Cow's milk is the major mean of MAP transmission from infected cattle to humans. In developed countries, cow's milk-based formulas are certainly a frequent source of exposure to exogenous complex proteins in postnatal life [37]. After 7 years of the TRIGR follow-up, no significant difference in progression towards *β*-cell autoimmunity was detected between children with HLA-conferred T1D susceptibility weaned to a conventional or an extensively hydrolyzed formula, the latter containing 99.7% of peptides with a molecular weight >2 kDa [27]. In our opinion, MAP, if present, may survive enzymatic treatment; nevertheless, MAP-peptides whose molecular weight ranges from 0,86 kDa for MAP3865c_125–133_ to 1,83 kDa for MAP3865c_125–133_ could persist in the hydrolyzed formula following enzymatic digestion and act as antigens stimulating T1D autoimmunity. Furthermore, breastfeeding continued during the study period could contribute to the transfer of MAP, MAP-derived peptides, or anti-MAP antibodies from mother to child. Macrophages are the most abundant cells in the breast milk counting 40–80% of cell fraction [38] and the primary target of MAP. Studies on cattle with subclinical and overt Johne's disease confirmed that MAP is shed into colostrum and milk during lactation upon its isolation in culture and by real-time PCR targeting IS900 MAP-specific gene [39]. Similarly, MAP has been isolated from human breast milk of few Crohn's patients [40]; however, research is required to assess the coincidence of T1D onset with the presence of MAP in mother's breast milk and its transmission pathway to offspring.

MAP adherence to the mucosa lining the small intestine and the following uptake by M cells and enterocytes may play a triggering role in antibody production [41]. A contribution of MAP in inducing a latent infection in humans could result from molecular mimicry with ZnT8 and PI epitopes, leading to autoimmune responses. Cross-reactivity to the common target sequences and specificity of the homologous anti-MAP/ZnT8 Abs verified by competition assays in previous studies [8] demonstrated recognition of a transmembrane domain of ZnT8 protein that cannot be evaluated by standard anti-ZnT8 Abs tests which employ a fusion protein combining extraluminal domains. The same ZnT8 region has a high homology with HP-derived peptides already evaluated in association with autoimmune thyroiditis [42]; in contrast to MAP, anti-HP Abs were detected at very low levels with similar prevalence in children at risk for T1D and the control group providing additional support for the association of MAP with autoimmune diabetes; therefore, responsiveness to MAP-derived antigens in the former group cannot be explained by an increased overall immune reactivity. A high degree of correlation between the homologous peptides further points at their cross-reactivity and segregation within the same sera.

Even though anti-IAA is considered the first circulating antibody to activate islet cell autoimmunity [43] and the single *β*-cell-specific autoantigen in postnatal period, birth cohort studies revealed that IAA can be detected only from 6 months of age in children genetically predisposed for T1D [3]. In our study, all human peptides and their MAP homologs were detectable during the first 6 months after birth in 2 out of 6 positive children for which samples collected right after delivery were available. Response against insulin in three ARS partially followed positivity to the proinsulin peptides. One subject developed an early immunity against both PI peptides and their MAP homologs; regardless of missing anti-PI Abs in the other two children, although anti-MAP Abs were present in one of them, progression to diabetes was observed exclusively in these three cases (13,04%) during sample collection period and was accompanied by multiple islet autoimmunity. Two subjects positive for all the assessed islet autoantibodies were younger at T1D onset (17-18 months) compared to the subject with IAA, IA2A, and ICA negative for GADA (4 years). Children positive for ICA as a single autoantibody recognized at least five anti-MAP and the homologous peptides.

In the majority of cases positive for the 8 analyzed peptides, seroreactivity to most of the epitopes appeared at the same time-point. In contrast, children displaying incomplete responses to MAP, ZnT8, and PI peptides presented an Abs pattern with gradual detection in the space of years. Persistence of peptide Abs and its duration occurred more frequently along with multiple positivity. From the three cases of T1D onset, two were positive for at least one anti-MAP Ab detectable from 6 months to one year prior to *β*-cell autoimmunity. In other children, this gap was even longer, ranging from 2 to 8 years. The lack of response to any of the investigated peptides in the child who progressed to diabetes may be explained by a multiple etiology of T1D and is reflected by our previous findings describing high but not total prevalence of anti-MAP Abs in diabetes patients [8–10]. One possibility may be that not all children develop Abs against peptides described in this study before overt diabetes.

The present study has a few limitations. We avoided describing Sardinian control subjects as the relative data have been already published [9]. In this case, prevalence of antibodies against the same MAP/ZnT8 homologous pairs reached similar values as those obtained in controls from continental Italy: 6,7% versus 0–9%. Moreover, mean age of Sardinian controls (8,3 years) was higher compared to the controls used in the current study (4,3 years); the latter one matched better our purpose to evaluate early responses to MAP-derived epitopes in children at risk for T1D starting from samples collected at birth. Unfortunately, HLA genotype was not available for controls since they were collected for other purposes. Similarly, individual formula treatment assignments cannot be disclosed due to the ongoing follow-up. Individual clinical data relative to the progression of diabetes-related autoimmunity following TRIGR's 7-year follow-up period are not revealed. Furthermore, samples covering the entire age range were not available for every participant, leading to the loss of an interesting subject with early response to all the analyzed peptides; lack of samples collected in the first months after birth for some children, including those who developed T1D, made the early Abs pattern impossible to evaluate ultimately. To complete the cross-reactivity of MAP and the homologous ZnT8/PI peptides investigated at antibody level, evaluation of T-cell responses would give an insight into early events in the autoimmune cascade due to CD4^+^ T-cells followed by autoreactive CD8^+^ T-cells. Provided a limited number of subjects are involved in this study, further investigation is necessary to achieve better understanding of mechanisms through which MAP could contribute to T1D development and a statistical significance of the analyzed data. In addition, more children with insulin autoantibodies as well as positivity to the extraluminal ZnT8 region should be evaluated in order to assess a possible link with the corresponding proinsulin and ZnT8 peptides. Upon termination of the TRIGR study (i.e., when the youngest participant will turn 10 years old), a correlation with diet may reveal whether positivity to ZnT8, PI, and homologous MAP peptides in our samples is significantly associated with formula type.

## Figures and Tables

**Table 1:** Demographic characteristics and pattern of islet autoantibodies in T1D at-risk subjects.

ID	Age at first blood collection (months)	Age at detection of islet Abs (months)	ICA	IAA	GADA	IA2A
2	4	—	−	−	−	−
3	0	—	−	−	−	−
4	3	—	−	−	−	−
**5**	3	17	+	+	+	+
6	0	—	−	−	−	−
8	0	—	−	−	−	−
**9**	6	48	+	−	−	−
**10**	3	97	+	−	−	−
**12**	24	48	+	+	−	+
**14**	11	18	+	+	+	+
16	24	—	−	−	−	−
**20**	24	85	+	−	−	−
22	18	—	−	−	−	−
24	9	—	−	−	−	−
26	0	—	−	−	−	−
27	12	—	−	−	−	−
32	24	—	−	−	−	−
33	12	—	−	−	−	−
34	0	—	−	−	−	−
38	0	—	−	−	−	−
39	0	—	−	−	−	−
40	0	—	−	−	−	−
43	0	—	−	−	−	−

0: blood collected at birth.
